# Editorial: Food-polyphenol-induced modulation of neurodegeneration

**DOI:** 10.3389/fnut.2023.1234550

**Published:** 2023-07-04

**Authors:** Thea Magrone, Omar Cauli, Rodrigo Wladimir Valenzuela

**Affiliations:** ^1^Microbiology and Virology Unit, Azienda Ospedaliero Universitaria Consorziale Policlinico di Bari, Bari, Italy; ^2^Nursing Department, University of Valencia, Valencia, Spain; ^3^Department of Nutrition, Faculty of Medicine, University of Chile, Santiago, Chile

**Keywords:** polyphenols, human studies, animal studies, neurodegenerative disorders, neuroprotection

Neurodegenerative diseases are continuously increasing due to a number of factors, such as emotional stress, socio-economic conditions, lifestyles such as alcohol abuse, smoking, antibiotic abuse, malnutrition, and excessive intake of junk food, as well as a lack or poor physical activity, all of which lead to several diseases such as diabetes, cardiovascular diseases, and cancer as well as a significant alteration of the homeostasis of the gut microbiota. Therefore, regarding neurodegenerative diseases, the proper function of the so-called gut microbiota-brain axis, where a bidirectional communication affecting other systems such as the neuroendocrine system, the enteric nervous system, the autonomic nervous system, and the immune system, is of great importance. During a process of intestinal dysbiosis, an altered permeability of the blood-brain barrier (BBB) takes place in which certain metabolites of the intestinal microbiota, which contains a vast variety of bacteria, viruses, and fungi, cross into the central nervous system, leading to neuroinflammatory processes also caused by an access of cells of the immune system which produce pro-inflammatory mediators. In Parkinson's disease (PD), for example, alteration of the gut microbiota would lead to the passage of lipolysaccharides (LPS) from the outer membrane of Gram-negative bacteria into the bloodstream and BBB. Microglia possess Toll-like receptor 4, which can recognize and bind to LPS, thus triggering the activation of nuclear factor kappa-light-chain-enhancer of activated B cells (NF-κB), which, in turn, initiates the transcription of pro-inflammatory cytokines and nitric oxide, thus leading to neuroinflammation. Furthermore, LPS would give rise to the mitochondrial accumulation of α-synuclein with a subsequent increase in reactive oxygen species and reactive nitrogen species resulting in functional alterations within mitochondria. Moreover, mitochondrial accumulation of α-synuclein promotes damage to dopaminergic neurons due to reduced dopamine production (1, 2).

Polyphenols are abundantly distributed in fruit and vegetables and their ingestion through food represents a healthy approach to prevent or delay several diseases due to their beneficial effects, which include anti-allergic, antioxidant, anti-cancer, and anti-neurodegenerative properties (3). In relation to neurodegenerative diseases, these natural compounds can, through different mechanisms, reduce and/or improve several conditions such as age-associated cognitive decline and schizophrenia (Figure 1).

**Figure 1:**
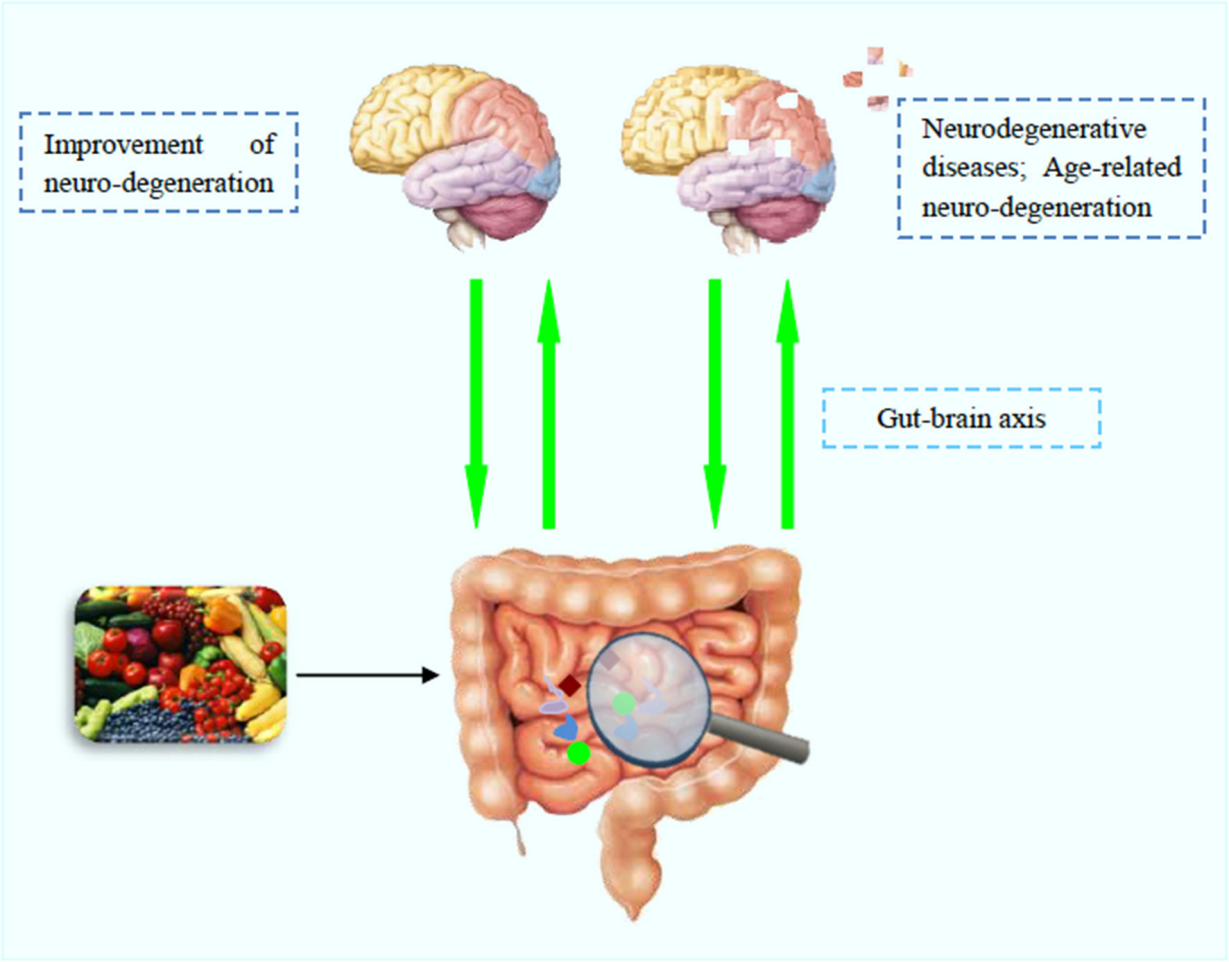
Polyphenols' beneficial effects in brain function and neurodegenerative diseases.

Li C. et al. have reported the effects of the intake of green tea rich in polyphenols on PD progression through a two-sample Mendelian randomization (MR) study using genome-wide association data from individuals affected by PD; they showed that green tea intake exerted a protective effect against dementia and depression associated with PD. In addition, the authors performed other statistical analyses such as the random effects inverse variance weighted method, MR Egger regression, weighted median and weighted mode, and MR analysis pleiotropy residual sum and outliers, which confirmed their obtained results.

Li W. et al. performed a 1-year follow-up study recruiting elderly Japanese individuals who were submitted to different tests including magnetic resonance imaging. In addition, a questionnaire was administered where participants recorded the type of tea they consumed and how often they drank it. Other information regarding gender, education, smoking, and physical activity were recorded. The investigations were conducted on two groups of individuals. One group included individuals without cognitive impairments while the other group had cognitive impairments. MRI revealed that elderly subjects who regularly drink tea have a lower risk of cognitive decline and a correlation with the volume of the posterior corpus callosum has been observed. In summary, consumption of tea rich in polyphenols would be able to reduce memory decline and associative learning.

Arozal et al. have evaluated the neuroprotective effects exerted by *Moringa oleifera* seed oil (MOO) and aqueous extracts of *Moringa oleifera* leaves (MOEs), both rich in polyphenols, on male BALB/c mice that were injected with scopolamine, an anticholinergic drug. BALB/c mice were subdivided into four groups: the control group, the scopolamine group who were injected the scopolamine, the MOO plus scopolamine groups, and the MOEs plus scopolamine group. Behavioral studies, Y maze test, and NOR test were carried out before animals were sacrificed. Post-mortem plasma and hippocampus were collected to evaluate the total antioxidant capacity (TAC) and to evaluate acetylcholine esterase activity, NF-κB, and Brain-Derived Neurotrophic Factor (BDNF) expression. Results obtained showed that both MOO and MOEs in the presence of scopolamine did not increase serum TAC levels. Furthermore, in the scopolamine-treated group, the expression of NF-κB was increased, thus provoking a neuro-inflammatory process and a cognitive deficit, whereas in the MOO plus scopolamine group the acetylcholinesterase activity was increased. Finally, the group of mice treated with scopolamine alone showed an upregulation of the expression level of tropomyocin receptor kinase B, the BDNF receptor. Pretreatment with MOO also significantly improved the expression of this protein. Thus, MOO may ameliorate the short-term memory impairment induced by scopolamine.

Liang et al. have demonstrated the ability of paeoniflorin (PF) to inhibit schizophrenia-like behavior induced by MK-801. The study was carried out using male BALB/c mice that were administered intraperitoneally with MK-801 and, following subdivision into groups, these animals were administered with paeoniflorin at different dosages whereas only one group was administered with paeoniflorin plus olanzapine. After drug treatments, all groups of animals underwent behavior tests. An *in vitro* study using primary cultured hippocampal neurons treated with PF at different concentrations was also conducted. Superoxide dismutase and malondialdehyde activity were measured in the serum of all animal groups and in the culture medium of hippocampal neurons. Data obtained showed that PF decreased oxidative stress due to increased levels of superoxide dismutase and reduced levels of malondialdehyde in MK-801-treated mice also inhibited the expression of pro-apototic protein Bax.

In conclusion, the results obtained from experimental studies in humans and animals have shown that the use of polyphenols could represent new therapeutic approaches for several pathological conditions affecting the central nervous system. Although further research needs to be conducted, this Research Topic may allow readers a clearer understanding about the pleiotropic role played by polyphenols.

## Author contributions

All authors listed have made a substantial, direct, and intellectual contribution to the work and approved it for publication.

## References

[1] Magrone T Magrone M Russo MA Jirillo E. Peripheral immunosenescence and central neuroinflammation: a dangerous liaison-a dietary approach. Endocr Metab Immune Disord Drug Targets. (2020) 20:1391–411. 10.2174/1871530320666200406123734 32250234

[2] Magrone T Marzulli G Jirillo E. Immunopathogenesis of neurodegenerative diseases: current therapeutic models of neuroprotection with special reference to natural products. Curr Pharm Des. (2012) 18:34–42. 10.2174/138161212798919057 22211682

[3] Magrone T Magrone M Russo MA Jirillo E. Recent advances on the anti-inflammatory and antioxidant properties of red grape polyphenols: *in vitro* and *in vivo* studies. Antioxidants. (2019) 9:35. 10.3390/antiox9010035 31906123 PMC7022464

